# Artificial Intelligence and Early Detection of Breast, Lung, and Colon Cancer: A Narrative Review

**DOI:** 10.7759/cureus.79199

**Published:** 2025-02-18

**Authors:** Omofolarin Debellotte, Richard L Dookie, FNU Rinkoo, Akankshya Kar, Juan Felipe Salazar González, Pranav Saraf, Muhammed Aflahe Iqbal, Lilit Ghazaryan, Annie-Cheilla Mukunde, Areeba Khalid, Toluwalase Olumuyiwa

**Affiliations:** 1 Internal Medicine, Brookdale Hospital Medical Center, One Brooklyn Health, Brooklyn, USA; 2 Internal Medicine, Landmark Medical Center, Woonsocket, USA; 3 Medicine and Surgery, Ghulam Muhammad Mahar Medical College, Sukkur, PAK; 4 Internal Medicine, SRM Medical College Hospital and Research Centre, Chennai, IND; 5 General Medicine, Clínica Renovar, Villavicencio, COL; 6 Internal Medicine, SRM Medical College and Hospital, Chennai, IND; 7 Internal Medicine, Muslim Educational Society (MES) Medical College Hospital, Perinthalmanna, IND; 8 General Practice, Naseem Medical Center, Doha, QAT; 9 Medicine, Yerevan State Medical University, Yerevan, ARM; 10 Internal Medicine, Escuela de Medicina de la Universidad de Montemorelos, Montemorelos, MEX; 11 Respiratory Medicine, Sikkim Manipal Institute of Medical Sciences, Gangtok, IND; 12 Medicine, Allsaints University School of Medicine, Roseau, DMA

**Keywords:** artificial intelligence, artificial intelligence in medicine, breast cancer detection, colon cancer detection, lung cancer detection

## Abstract

Artificial intelligence (AI) is revolutionizing early cancer detection by enhancing the sensitivity, efficiency, and precision of screening programs for breast, colorectal, and lung cancers. Deep learning algorithms, such as convolutional neural networks, are pivotal in improving diagnostic accuracy by identifying patterns in imaging data that may elude human radiologists. AI has shown remarkable advancements in breast cancer detection, including risk stratification and treatment planning, with models achieving high specificity and precision in identifying invasive ductal carcinoma. In colorectal cancer screening, AI-powered systems significantly enhance polyp detection rates during colonoscopies, optimizing the adenoma detection rate and improving diagnostic workflows. Similarly, low-dose CT scans integrated with AI algorithms are transforming lung cancer screening by increasing the sensitivity and specificity of early-stage cancer detection, while aiding in accurate lesion segmentation and classification.

This review highlights the potential of AI to streamline cancer diagnosis and treatment by analyzing vast datasets and reducing diagnostic variability. Despite these advancements, challenges such as data standardization, model generalization, and integration into clinical workflows remain. Addressing these issues through collaborative research, enhanced dataset diversity, and improved explainability of AI models will be critical for widespread adoption. The findings underscore AI's potential to significantly impact patient outcomes and reduce cancer-related mortality, emphasizing the need for further validation and optimization in diverse healthcare settings.

## Introduction and background

**Figure 1:**
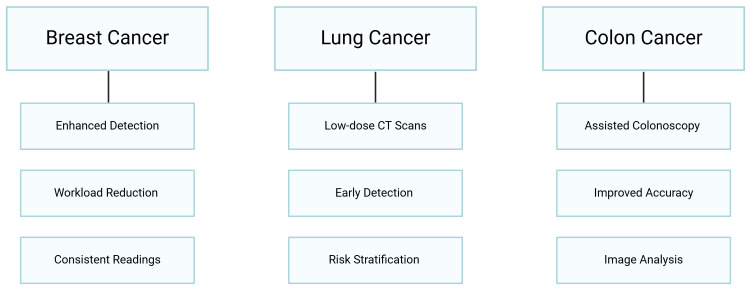
Role of Artificial intelligence in cancer screening (Image credit: Rinkoo FNU)

Early detection of cancer has been, and continues to be, one of the most significant challenges in the field of medicine over the years. Screening strategies have been implemented, significantly improving patient outcomes and survival by detecting tumors at a more treatable stage [1, 2]. In 2022, almost 20 million new cancer cases were detected, and 9.7 million individuals succumbed to the illness globally [3]. In 2022, over 1.9 million new cancer cases are projected to be diagnosed, with 609,360 cancer-related fatalities in the United States [4]. Colorectal, lung, and breast cancers are among the most common and lethal forms of cancer worldwide, in which these screening programs have had a significant impact [5-7]. However, these screening programs often face resource limitations, diagnostic variability among radiologists, and patient accessibility (Figure 1) [8, 9].

In recent years, artificial intelligence (AI) has emerged as a promising tool to enhance cancer screening programs, offering the potential to address some of these limitations. AI, mainly through advancements in machine learning and deep learning algorithms, has shown remarkable capabilities in medical image analysis, predictive analytics, and decision support [1, 9]. AI-powered systems can assist radiologists in identifying subtle patterns in imaging data, assist endoscopists in optical diagnosis of colorectal polyps, and much more, thus, reducing false-positives and -negatives and prioritizing high-risk patients for further investigation. However, despite these advancements, challenges such as data bias, lack of model transparency, and difficulties in integrating AI systems into clinical workflows remain significant barriers. Addressing these challenges is crucial to ensure the reliable and equitable application of AI in cancer screening [10-12].

This narrative review delves into the current and potential applications of AI in colorectal, lung, and breast cancer screening programs. It underscores how AI, by enhancing diagnostic accuracy and streamlining workflows, can significantly contribute to better patient outcomes. However, beyond these benefits, this review also examines critical challenges such as data quality, accessibility, and the complexities of clinical implementation. By addressing these limitations alongside the advantages, this review aims to provide a comprehensive overview of the opportunities for and barriers to leveraging AI for the early detection of these cancers.

## Review

Breast cancer

Breast cancer has been reported as a significant health issue. It is the most commonly diagnosed form of cancer among women globally and the primary cause of mortality linked to female cancers [13]. While there have been advancements in the detection and treatment of breast cancer [13], the mortality rates are still concerning [14]. Bray et al. estimate that annually, more than 500,000 women lose their lives to breast cancer across the globe [13]. Detecting breast cancer early and responding promptly play an instrumental role in lengthening life expectancy [14, 15]. Studies indicate that incorporating AI can potentially enhance the effectiveness of these methods in detection [16-18].

Early Breast Cancer Detection Using Deep Learning Techniques

Convolutional neural network (CNN) is a deep learning model used in AI that assists in image processing and grid-based data operations [19]. It excels in recognizing patterns within images like shapes and textures [19]. Gated recurrent unit (GRU) is a form of deep learning model that addresses the limitations of CNN by enabling the model to preserve information over time and across various parts of the image leading to better detection of cancerous tissues [16].

Deep CNNs are extensively utilized in computer-aided detection (CADe) applications and are becoming more prevalent for lesion detection, risk assessment, image retrieval, and classification tasks in mammography. Mammography computer-aided design (CAD) systems can identify findings according to the BI-RADS lexicon (CADe) and classify lesions as benign or malignant (computer-aided diagnosis (CADx)) [20]. These systems are crucial in aiding radiologists' decision-making, reducing the time needed to assess a lesion, and decreasing the occurrence of false-positives that lead to unnecessary biopsies. Notable deep CNNs for mammogram classification include InceptionV3, DenseNet, ResNet50, VGGNet16, and AlexNet. The high accuracy of CNN results on mammograms offers a promising solution for more precise medical image detection. CNNs have demonstrated considerable promise in enhancing breast cancer screening, diagnosis, and classification. Despite the lengthy and data-intensive training process required for supervised CNNs, the outcomes are reliable and encouraging [20].

**Figure 2:**
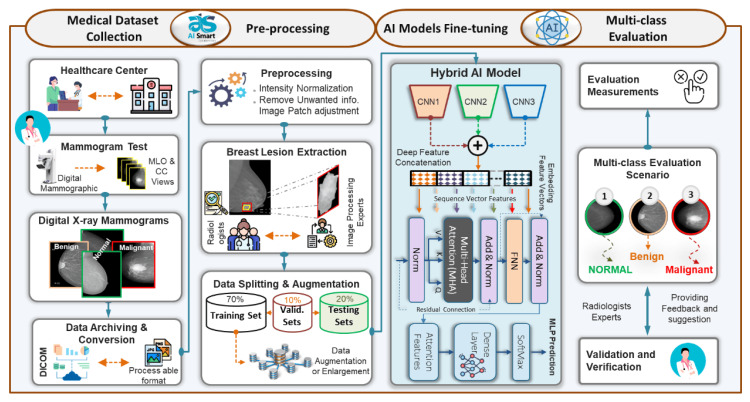
Using the ETECADx framework, we can differentiate between benign, malignant, and normal tissues around breast cancer lesions Reproduced from [18] under Creative Commons License.

The vision transformer encoder's (ViT) self-attention mechanism and ensemble transfer learning of CNNs are used to create ETECADx, an AI-based CAD system (Figure 2). The transformer encoder uses Approach A (binary classification) and Approach B (multi-classification) to diagnose breast cancer, while the backbone ensemble network produces precise and useful high-level deep features. The proposed CAD system uses the public benchmark multi-class INbreast dataset. Specialist radiologists also collect and interpret private breast cancer images to test the ETECADx platform. INbreast mammograms have good assessment accuracy, 98.58% for binary and 97.87% for multi-class. For multi-class and binary breast cancer prediction, the ensemble learning model beats backbone networks by 4.6% and 6.6%, respectively [21]. The hybrid ETECADx's prediction performance increases by 8.1% for binary diagnosis and 6.2% for multi-class diagnosis using the ViT-based ensemble backbone network. When evaluated using breast images, the proposed CAD system achieves 97.16% binary and 89.40% multi-class prediction accuracy. On average, ETECADx can detect breast abnormalities in 0.048 seconds in each mammogram. Such promising findings may strengthen practical CAD framework applications that combat breast cancer as a second line of defense [21].

In the study conducted, one of the significant performance metrics for the model included an 86.2% accuracy level, indicating its capability to differentiate effectively between both cancerous and non-cancerous tissues. This ensures that treatment can be started early while unnecessary anxiety by false-positives is minimized. The data also indicated an 85% precision level for instances identified as invasive ductal carcinoma (IDC) positive by the model, which is crucial as precision rates help minimize false-positives and prevent individuals from undergoing unnecessary biopsies or treatments. The data also showed that 84.71% of cases without cancer were accurately identified as not having IDC. This finding is important as a high specificity rate indicates positives and can prevent unnecessary diagnoses for patients who do not have IDC. Lastly, the data showed a 0. 89 area under the curve (AUC) score, indicating that the model can distinguish between IDC and IDC cases at points of decision-making [16]. This finding is crucial, as the AUC value demonstrates the model's ability to effectively balance sensitivity and specificity in cancer diagnosis, enabling accurate detection without excessive false-positives. This insight is significant as it highlights the connection between AI and its potential to detect early-stage breast cancer.

Role of AI in breast cancer detection 

Early Breast Cancer Detection Utilizing Deep Learning: DE-Ada Model 

*DE-Ada* model utilizes a combination of techniques to improve the categorization of breast masses [17]. It analyzes mammography datasets from the Curated Breast Imaging Subset of Digital Database for Screening Mammography (CBIS-DDSM) and INbreast databases for classification based on attributes like shapes and textures of breast masses. The CBIS-DDSM dataset has 22,145 images. The exact size of the INbreast dataset is unknown. It utilizes elements from methods, like scale-invariant feature transform (SIFT) and visual geometry group (VGG), alongside histogram of oriented gradients (HOG) to improve precision in classifying performance across different datasets. The DE-Ada model ensures generalizability and accuracy in breast cancer detection by integrating diverse feature extraction techniques like SIFT, VGG, and HOG for robust classification. It enhances adaptability through cross-dataset validation using the CBIS-DDSM and INbreast, along with data augmentation and transfer learning to mitigate dataset biases. Additionally, techniques like synthetic minority over-sampling technique (SMOTE) and adaptive learning improve model performance across varying data distributions, ensuring reliable early detection of breast cancer. [17].

**Figure 3:**
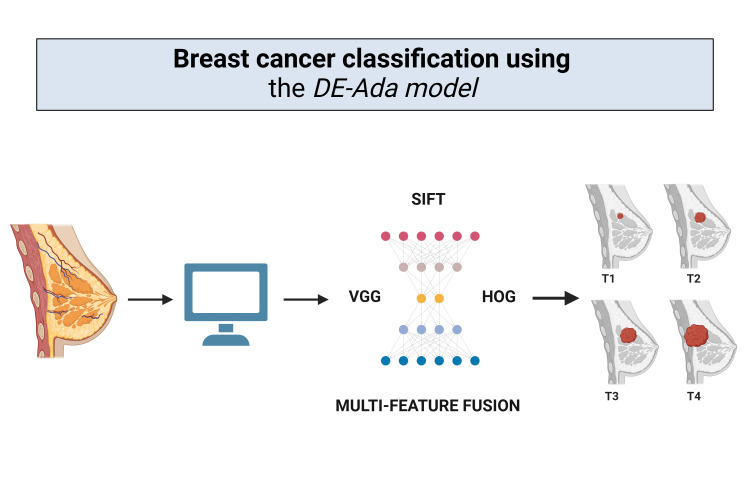
Early Breast Cancer Detection Utilizing Deep Learning: DE-Ada Model (Image credit: Omofolarin Debellotte)

Discovering patterns in raw data is a common use case for deep learning. Detecting breast cancer has become more prevalent in recent decades using deep learning. As much as a year ahead of schedule compared to traditional clinical approaches, deep learning algorithms may detect breast cancer. Numerous deep learning-based strategies, such as CNN, recurrent neural network, deep neural network, and autoencoder (AE)-based methodologies, have been developed for the purpose of breast cancer detection [22]. Train a deep neural network to generate posterior distributions for all possible values by modelling the input distribution with its encoding. The matrix logic predictor is an example of a feed-forward network. AE's output layer is a generative model that uses input data to reproduce itself. One method of deep learning that is generatively semi-supervised is generative adversarial network (GAN) [22].

Deep Learning in Digital Breast Tomosynthesis for Breast Cancer Detection

One such technique that has revolutionized breast imaging is digital breast tomosynthesis (DBT). The use of DBT, a kind of 3D mammography, is quickly superseding that of the more conventional 2D mammography. One benefit of DBT over conventional 2D mammography is its ability to detect tiny abnormalities that would otherwise go undetected [23]. Cancerous lesions may be identified using the Breast Imaging - Reporting and Data System by the use of asymmetry, bulk, microcalcifications, or architectural deformation. Compared to 2D imaging, 3D imaging excels at spotting deformities that are difficult to annotate. The use of AI-CAD systems in DBT has the potential to uncover diagnoses that were previously undetectable by conventional mammography [23].

*Deep Learning-Based CAD Systems for Breast Cancer Diagnosis* 

AI in breast cancer (BC) diagnosis can serve as a second opinion for radiologists, aiding in making accurate decisions where misdiagnosis must be avoided. AI integration aids in distinguishing between suspicious and more suspicious breast lesions. AI advancements have enhanced the capabilities of computer-aided diagnosis (CADx), expediting both diagnosis and treatment. AI-assisted diagnosis provides several benefits: (1) It's minimally invasive, reducing the need for biopsies; (2) It can distinguish molecular features to classify subtypes; (3) It can identify the heterogeneity of breast lesions; and (4) It can analyze tumor progression or treatment response [24]. While AI-based diagnosis supports radiologists in decision-making, it cannot replace independent imaging and clinical diagnosis. The use of AI in breast cancer detection has reduced the number of false-positives and incorrect diagnoses made by radiologists. AI offers a dispassionate evaluation that takes into account internal structure, texture, unique characteristics, and more for accurate diagnosis and categorization. There are a few drawbacks to AI-based diagnosis. Firstly, there isn't enough generalization in AI methodologies to produce repeatable results. Secondly, algorithms that can handle image data from different modalities and patient-independent variations need to have low false-positive rates and high specificity [24]. Lastly, prior to clinical practice adoption, real-time clinical trial validation on a large sample size is necessary. Microcalcification clusters, dense tissue lumps, architectural deformities, questionable mass margins, and dense tissue structure in mammograms may all be seen and highlighted using X-ray-based CAD systems. It is common practice to augment X-ray mammography with magnetic resonance imaging or ultrasound in order to better detect thick, difficult-to-compress breast tissue in some individuals. Having said that, that is not so with mammography. If a patient is too sick to receive an MRI or if a pregnant woman should not be subjected to X-rays, an ultrasound (US) of the breast is the best alternative [24].

The results for autonomous AI's AUC ranged from 0.81 to 0.97, reflecting a strong overall diagnostic performance across studies. The only simulation study that directly compared radiologists to AI alone found that the AI’s performance was comparable to that of human radiologists. This was demonstrated by the AUC difference of 0.03, with the 95% confidence interval for this difference not falling below 0.05. This suggests that the performance gap between radiologists and AI systems is minimal, indicating that AI has the potential to serve as a robust diagnostic tool. However, it is worth noting that while AI systems match radiologists in terms of overall AUC, their diagnostic behavior may differ in terms of sensitivity and specificity, depending on the clinical application and dataset used [25]. Researchers found mixed results when comparing radiologists and AI systems on their own; AI systems showed either better or worse accuracy, or lower specificity with slight gains in sensitivity. Fitted summary receiver operating characteristic (sROC) curves demonstrate a little gain in the AUC that matches the magnitudes of improvements seen at the study level, and research comparing radiologists to radiologists boosted with AI consistently shows better accuracy for the latter [25]. Given the marginal increase in sensitivity, the cost-effectiveness and number needed to treat (NNT) benefits of AI implementation remain uncertain.

Recent iterations of Transpara exhibit lower false-negative rates compared to an earlier version, suggesting that advancements in AI have decreased the chances of undetected cancers. Nonetheless, there has been a rise in false-positive rates, implying that increased AI sensitivity might result in more recalls and errors [26]. The analysis also emphasizes the need for a proper reference standard to categorize false-positives and negatives. Research including both screen-detected and symptomatic cancers showed a reduced false-positive rate for AI than those with only screen-detected cancers, reflecting the limitations of the latter in confirming true positive AI findings dismissed by radiologists [26]. AI's oversight of interval cancers also led to elevated false-negative rates in such studies. The lack of comprehensive interval cancer data has been recognized as a potential bias in AI studies with empirical research indicating inflated accuracy rates.

Challenges in AI for breast cancer screening 

Variability in AI Detection 

As stated above, the *DE-Ada* model utilizes a combination of techniques to improve the categorization of breast masses. It analyzes mammography datasets from CBIS-DDSM and INbreast databases for classification based on attributes like shapes and textures of breast masses [17].

Sensitivity is a critical metric in cancer detection, as it measures how accurately a model identifies true positive cases, ensuring early diagnosis and timely treatment. In this study, the AI model demonstrated varying sensitivity across different datasets. The sensitivity of the model was 82.96% on the CBIS-DDSM dataset, indicating strong performance in detecting cancer cases, which is crucial for minimizing missed diagnoses. However, on the INbreast dataset, the sensitivity dropped to 57.20%, suggesting potential limitations in detecting certain cancer cases. This disparity highlights the variability in model effectiveness across datasets and underscores the need for further optimization to improve detection rates in diverse clinical scenarios [17]. 

Operator Expertise and Systemic Evaluation of Algorithms

There are numerous commercial AI systems available, and while some are more accurate than others, their performance is influenced by factors such as the user's specific demographic and equipment, the system's intended application, and the deployment possibilities.

AI technology has the potential to enhance breast cancer screening by increasing accuracy and lowering radiologist effort. Deep learning-based AI systems show potential in boosting detection performance and minimizing variability among observers. Standardized norms and trustworthy AI procedures are important to assure fairness, traceability, and robustness. Further study and validation are required to create clinical confidence in AI. A collaboration between researchers, physicians, and regulatory agencies is vital to overcome difficulties and encourage AI application in breast cancer screening [27]. 

Breast cancer is the most commonly diagnosed cancer and the second leading cause of mortality among women, with approximately one in eight U.S. women (13%) developing invasive breast cancer in their lifetime. Early detection improves survival rates and reduces treatment costs. Imaging techniques like mammography, CT, MRI, PET/CT, and histopathology aid diagnosis but depend on expert analysis, which is costly and error prone. Deep learning (DL) has shown promising results, achieving high sensitivity, specificity, and AUC scores in retrospective studies. However, external validation is necessary for clinical adoption. This research reviews existing studies, datasets, and key challenges, highlighting future AI applications in breast cancer detection [28].

Clinical Integration 

A further challenge with AI is winning over doctors' trust. Many medical professionals lack the trust necessary to depend on AI when making decisions [29]. Radiologists are finding it difficult to work with AI due to their concerns that the technology may one day take over some of their duties. Doctors should have extensive education on how to utilize AI for breast cancer diagnosis. The radiologist may use it as a tool to assist in diagnosis and decision-making. Its noninvasive nature makes it a valuable option, and further research could enhance its effectiveness by integrating more advanced AI capabilities [29].

Colon cancer

Al Techniques and Technologies in Colon Cancer Screening 

**Figure 4:**
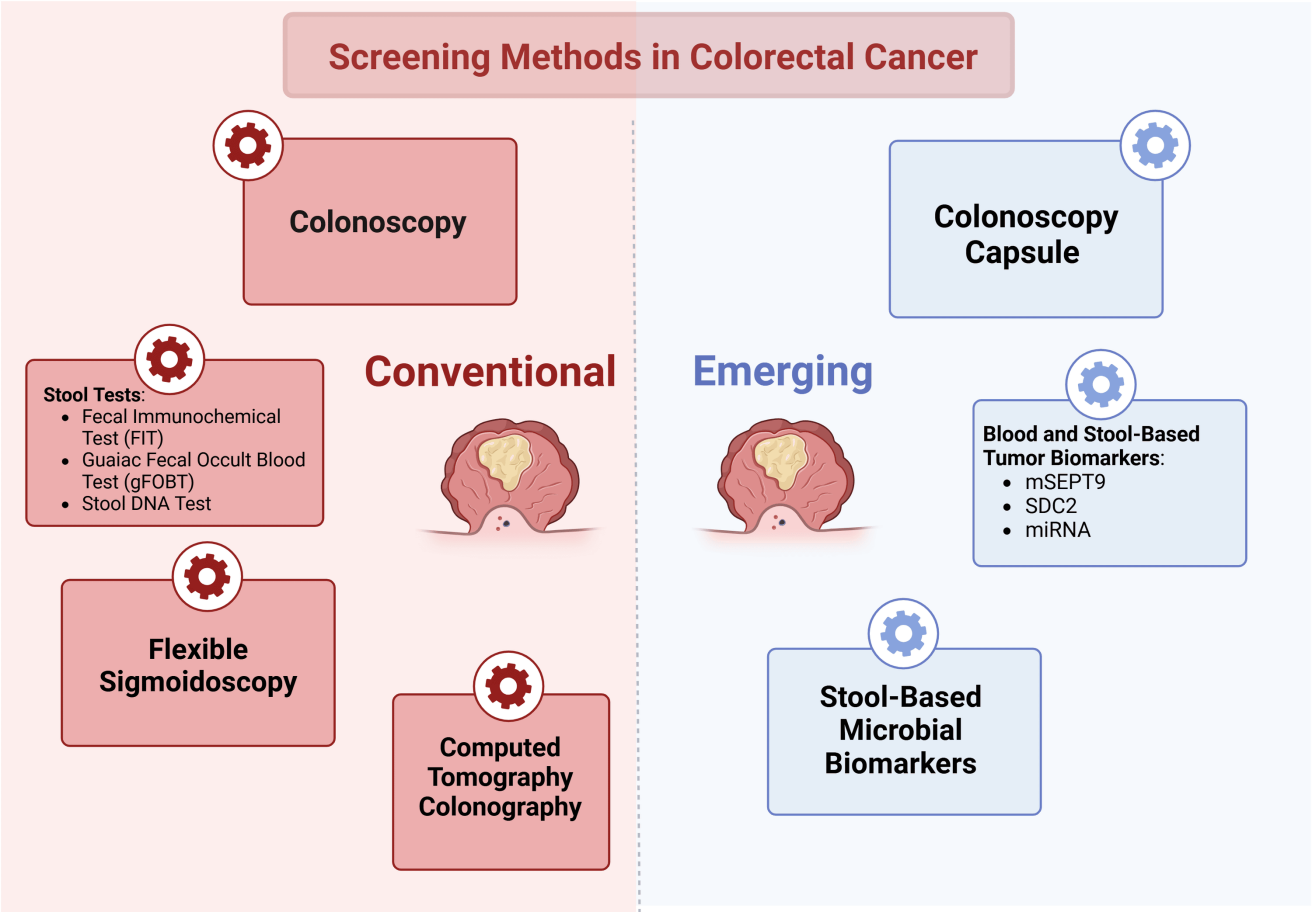
Screening methods for colon cancer (Image credit: Omofolarin Debellotte)

Artificial intelligence includes machine learning (ML) and deep learning (DL). ML and DL contribute to lesion localization, reducing misdiagnosis rates, and enhancing diagnostic accuracy (Figure 4) [30].

Large datasets are stored using ML, which is then utilized to train prediction models and derive generalizations. ML is a set of computational approaches that improves our understanding of disease outcomes by making use of visual features acquired from radiomics. Unsupervised and supervised ML are the two main approaches in radiomics. When it comes to classifying information, unsupervised ML doesn't rely on any prior data or data extracted from the image itself. Supervised ML, on the other hand, relies on an existing dataset to train the AI. The most recent subfield of ML, known as "deep learning", uses artificial neural networks to do image recognition and classification. A multi-layer neural network processes an image in DL, reducing it to a numerical representation of features for supervised ML algorithms [30].

CNNs are well-known in the field of DL for their ability to extract high-level information from similar components located in different parts of the input signal. This has led to CNN's impressive performance in both visual and speech recognition tasks, with CNN particularly shining in the former due to its exceptional picture processing capabilities [30].

CADe and CADx

Computer-aided detection (CADe) is designed to assist endoscopists in identifying more, faster, and smaller adenomas during endoscopy. Computer-aided diagnosis (CADx) leverages the appearance of polyps or adenomas to predict their histopathological architecture, thereby accelerating the determination of appropriate treatment [31]. While CADe aims to decrease the rate of missed polyps during colonoscopy and ultimately increase the performance of the endoscopists, CADx has the property of real‐time interpretation of the polyp optical diagnosis, potentially being able to reduce the rate of unnecessary polypectomies of non‐neoplastic lesions [32]. CAD models demonstrate high diagnostic accuracy in predicting the histology of small colorectal polyps, boasting a pooled AUC of 0.96, a sensitivity of 0.93, and a specificity of 0.87. The diagnostic odds ratio stands at 87, reflecting a strong performance. Additionally, the negative predictive value for adenomatous polyps in the rectosigmoid colon is 0.96, exceeding the diagnostic thresholds necessary for informed decision-making [33].

*Recent Advancements in AI for Colonoscopy* 

Using AI-based tools during colonoscopy improves the adenoma detection rate by 30-50%, reduces variability by increasing consistency, and minimizing human error. It also has a lower missed adenoma rate, which makes it an excellent screening tool [34]. The AI tools used were computer-aided detection systems, which highlighted suspicious polyps, thus showing endoscopists potential cancerous lesions in real-time. Computer-aided diagnosis systems characterize lesions to determine if they are benign, precancerous, or cancerous. ML algorithms also characterize detected polyps based on large databases of polyp images. The last tool was real-time analysis, which analyzes, compares, and detects polyps on the colonoscopy live feed with its database to alert the clinician in a timely manner [35]. AI had detected precancerous polyps with an accuracy over 90% compared to chromoendoscopy and image-enhanced endoscopy [36]. Colonoscopy with AI significantly increased adenoma detection rates and polyp detection rates compared to colonoscopy without AI, with high certainty. Adenoma detection rate (ADR) with AI was 29.6% versus 19.3% without AI, and polyp detection rate (PDR) was 45.4% with AI versus 30.6% without AI, both showing a relative risk of around 1.5. There was no difference in the detection of advanced adenomas between the two groups, but mean adenomas detected per colonoscopy was higher for small adenomas (≤5mm) with AI compared to non-AI [37]. The AI system significantly increased the PDR (34.0% vs 38.7%, p < 0.001). AI-enhanced colonoscopy significantly outperforms conventional colonoscopy in ADR, as well as the speed and accuracy of polyp characterization, which could reduce costs associated with colorectal cancer by preventing unnecessary procedures [38]. AI can also help improve the quality of colonoscopy by assessing the effectiveness of bowel preparation before and during colonoscopy and predicting the depth of submucosal invasion [39].

Computer-aided detection and diagnosis were designed to help endoscopists detect more, faster, and smaller adenomas as well as real-time polyp characterization to increase true positive rates at a rate of 20% to 30% compared to endoscopy alone, the gold standard [31, 34, 40]. Endocytoscopy is an endoscopic technique, which consists of a contact light microscope placed at the tip of the colonoscope, that enables magnification of lesions as well as histologic characterization when combined with methylene blue staining. When endocytoscopy is combined with AI yields higher results than seasoned colonoscopists and even higher than trainees [41]. Chromoendoscopy is a modified endoscopy procedure that uses pigments, dyes, or stains to show mucosal patterns was tested against AI, and AI-enhanced colonoscopy detected adenoma with an accuracy above 90% [36].

AI-based detection of precancerous polyps has shown high accuracy, often exceeding 90%, and in some cases, matching expert-level performance [42]. Studies have indicated that AI-assisted colonoscopy improves the ADR compared to traditional white-light endoscopy and even some image-enhanced modalities [43].

AI can make rapid differentiation between neoplastic and non-neoplastic lesions, whereas chromoendoscopy requires additional staining and expert interpretation [44].

However, AI is not a complete replacement for advanced human-led endoscopic techniques. For instance, magnifying chromoendoscopy with crystal violet dye has been shown to reach 97.2% sensitivity, indicating that expert-performed chromoendoscopy can still offer superior histopathological prediction [44]. AI algorithms rely on large, high-quality databases for training. Any bias in the dataset (e.g., underrepresentation of certain polyp subtypes) can lead to reduced accuracy in clinical settings. A randomized controlled trial demonstrated that autonomous AI was as accurate as AI-assisted humans, but both exhibited only moderate accuracy (~77%) in optical diagnosis of polyps, suggesting that AI should not fully replace expert judgment [45].

Many AI algorithms function as "black boxes," making it difficult to understand why a particular polyp is classified as neoplastic or benign. This lack of transparency raises concerns about reliability and legal accountability [46]. AI performance varies depending on the type of imaging used (e.g., standard white-light imaging vs. chromoendoscopy). AI models trained on one imaging modality may not generalize well to another [47].

Sensitivity, Specificity, and Overall Diagnostic Accuracy of AI Models

Overall, AI systems have consistently been superior to conventional colonoscopy. For instance, GI-Genius and Medtronic have a sensitivity of 99.7%, a false-positive rate lower than one percent, and they were 82% faster in detecting adenoma than a visual endoscopic inspection. The CNN, a part of the DL models, showed a sensitivity, specificity, and accuracy for histologic diagnosis is 95.1%, 92.76%, and 93.48%, respectively [48].

A study compared the performance of endocytoscopy, described above, when combined with AI systems and experts as well as trainees. The sensitivity and specificity were 93% and 94%, respectively, for colorectal polyp detection by endocytoscopy with AI, whereas endocytoscopy performed by experts yielded a sensitivity of 90% and a specificity of 87%. Trainees yielded a sensitivity of 74% and a specificity of 72% [41].

Impact on Early Detection and Patient Outcomes

Early colorectal polyp detection means decreased colorectal cancer morbidity and mortality. The use of efficient screening tools is paramount in order to provide top-tier health care. Not only do good screening tools help detect abnormal polyps, but they also help decrease unnecessary polyp/adenoma resection [32, 36], avoiding biopsy-related medical complications as well as the costs it inquires for the hospital and the patients [36]. The conventional screening methods are stool tests: fecal immunochemical test and Guac fecal occult blood test, stool DNA test, flexible sigmoidoscopy, colonoscopy, and computed tomography colonography. The screening methods that are being developed are the colon capsule, blood and stool-based tumor biomarkers (mSEPT9, SDC2, miRNA), and stool-based microbial biomarkers. One aspect that is not conventionally taken into consideration is patient compliance. As far as colorectal cancer screening is concerned, bowel preparation may be the biggest obstacle for patients, so some researchers argue that blood and/or stool-based biomarkers can be better screening tools as they require minimal to no patient preparation [31].

Challenges for AI in Colon Cancer Screening 

Despite significant advancements in AI technologies, integrating AI-based systems into routine colon cancer screening faces several challenges. The accuracy of AI systems, such as CNN and computer-aided detection, varies widely depending on factors like the quality of datasets, equipment used, and the expertise of the medical practitioners involved. AI has shown potential in reducing adenoma miss rates and improving adenoma detection rates, yet numerous obstacles remain that limit its full adoption in clinical practice [48, 49]. AI's effectiveness in reducing adenoma miss rates and improving diagnostic accuracy has been well-documented in randomized controlled trials and systematic reviews [50-52]. While artificial intelligence holds great promise in improving colonoscopy outcomes, there are several inherent limitations in current AI systems that prevent widespread clinical adoption. These limitations stem from issues with diagnostic accuracy, integration into clinical workflows, and variability in both endoscopic equipment and practitioner expertise. CNN have emerged as a particularly effective method for detecting colorectal polyps and cancer. CNN-based models have shown promising results in terms of sensitivity and specificity when compared to traditional colonoscopy methods. However, the performance of CNNs is dependent on high-quality training data, and there is still variability in their diagnostic accuracy across different populations. A meta-analysis demonstrated that CNNs can achieve high diagnostic accuracy, but challenges related to overfitting and the need for larger, more diverse datasets remain [53].

Data-related Challenges

One of the foremost challenges lies in the quantity and quality of data used to train AI systems. High-quality, diverse datasets are necessary for AI models to generalize well across different patient populations. The limited availability of annotated medical datasets for colorectal polyps, particularly those representing rare lesions like sessile serrated lesions, hinders AI’s performance in real-world settings [48]. Most models rely heavily on image datasets derived from a single institution, raising concerns about the generalizability of AI models when applied to different clinical settings [54].

Real-world performance of AI-assisted colonoscopy was examined in a prospective randomized cohort study, where it was observed that AI tools significantly improved polyp detection rates. However, the study also highlighted challenges, such as the need for continuous updates to AI algorithms to account for new types of lesions and variations in patient populations. Despite improvements in ADR, the study emphasized the importance of further training for endoscopists to better collaborate with AI systems and to reduce workflow interruptions caused by AI-assisted diagnosis [55].

Diagnostic Accuracy

Although AI systems have shown improvements in ADRs and reducing adenoma miss rates, there are still limitations in terms of false-positives and false-negatives. False-positives may lead to unnecessary procedures and patient anxiety, while false-negatives can result in missed precancerous polyps, which undermine the purpose of AI in screening [49]. Studies have demonstrated that while AI systems improve detection, they are not yet flawless, and their accuracy often does not surpass that of highly skilled human endoscopists [48]. Furthermore, AI struggles particularly with certain types of lesions, such as sessile serrated lesions, which are more difficult to detect and have a high rate of being missed by both AI and human practitioners [54].

Integration into Clinical Workflow

Another major limitation is the challenge of effectively integrating AI systems into the existing clinical workflows. Colonoscopy procedures involve a complex set of decisions made by the endoscopist, and AI models need to be seamlessly incorporated into this decision-making process. However, AI systems often operate independently, leading to workflow interruptions and inefficiencies. Endoscopists may need additional training to understand and effectively interact with AI tools [50]. Moreover, there is a significant learning curve associated with using AI-based systems, which can deter some physicians from adopting these technologies in their practice [51].

Variability in Equipment and Operator Expertise

There is also variability in the effectiveness of AI systems due to differences in endoscopic equipment and operator skill levels. Studies have shown that AI models trained on data from specific high-end equipment may not generalize well to lower-cost or older systems commonly used in less resourced clinical environments [52]. Additionally, the expertise of the endoscopist plays a crucial role in how effectively AI is utilized during procedures. AI alone cannot compensate for a lack of technical skill in polyp detection and management, further limiting its current application in real-world settings [48, 52].

Future Directions for AI in Colon Cancer Screening

As AI continues to evolve, there are several key areas where improvements and advancements are needed to maximize its potential in colon cancer screening. Addressing these future directions can help overcome the existing challenges and limitations, ultimately leading to more accurate and efficient colonoscopy procedures.

One of the primary goals for future AI development is to improve the generalization of models across various clinical settings and patient populations. Current AI systems often perform well in controlled environments but struggle in diverse clinical settings due to a lack of broad data representation during training [49]. Expanding training datasets to include more varied images of colorectal polyps, especially from different institutions and equipment, will be crucial to improving model accuracy and applicability in real-world scenarios [48]. The collection of multi-institutional datasets and collaborative efforts between hospitals can contribute to this goal [54].

As AI systems become more integrated into clinical workflows, it will be important to improve their explainability. Explainable AI seeks to make the decision-making process of AI models more transparent, allowing endoscopists to understand how the AI arrived at a particular diagnosis. This can increase the trust and collaboration between AI and human practitioners [50]. By offering clearer insights into how diagnoses are made, AI can be viewed as a supportive tool rather than a black-box solution, thereby encouraging wider adoption in clinical practice [51]. 

For AI systems to gain widespread acceptance, there is a need for more long-term prospective studies that evaluate their effectiveness over time. These studies should focus on how AI impacts patient outcomes, such as long-term reduction in cancer incidence and mortality [52]. Additionally, external validation across various healthcare systems is essential to confirm the reliability and robustness of AI models. This will help bridge the gap between controlled research settings and the complexities of real-world applications [54]. 

The future of AI in colon cancer screening lies in collaborative systems where AI assists but does not replace the endoscopist. Co-decision models, where both the AI system and the clinician work together, could enhance diagnostic accuracy while maintaining human oversight [48]. AI should be viewed as an adjunct tool that provides additional insights, with final decisions being made collaboratively. This approach would help mitigate concerns about AI making autonomous decisions without adequate human involvement [51].

Lung cancer

Screening for Lung Cancer

Cancer screening involves administering standardized exams, tests, or procedures to a population in the hopes of detecting in asymptomatic persons cancer that has not yet been identified [56]. Although early-stage lung cancer often presents with subtle or no symptoms, approximately 70% of the cases are diagnosed at middle or late stages. The five-year survival rate for the advanced stage disease is a dismal 5.2%, while early detection can significantly improve the survival rate to 57.4% [57]. Much of the efforts are aimed at detecting disease earlier through effective screening so that patients can benefit from more treatment options and ultimately, a reduction in mortality. 

Several randomized controlled studies have shown that at-risk people were screened using chest X-rays and sputum cytology. While this led to early diagnosis, it did not reduce cancer-related death [58-61]. The promise of Ct for an early detection of lung cancer generated significant interest among clinicians upon its introduction to clinical practice. Traditional CT scans, with their extensive scanning times and significant radiation exposure (7 millisieverts), made this impossible. The significant advancement was the development of low-dose CT, which, although exposing patients to just 1.6 millisieverts of radiation, could yet create high-resolution pictures that were just as sensitive and precise as traditional CT scans for identifying lung nodules [62, 63]. This advancement has enabled the implementation of low-dose computed tomography (LDCT) for lung cancer screening. One successful strategy for lowering lung cancer death rates is LDCT screening [64]. Adults between the ages of 50 and 80 with a 20 pack-year smoking history, who smoke now or have stopped within the last 15 years, should get a lung cancer screening with LDCT once a year, according to the current recommendation from the United States Centers for Medicare and Medicaid Services. Problems include heavy workloads and high false-positive rates, which may cause patients undergoing needless treatments when recommendations are expanded and more nations use this screening technique.

AI and Lung Cancer Screening in CTs

**Figure 5:**
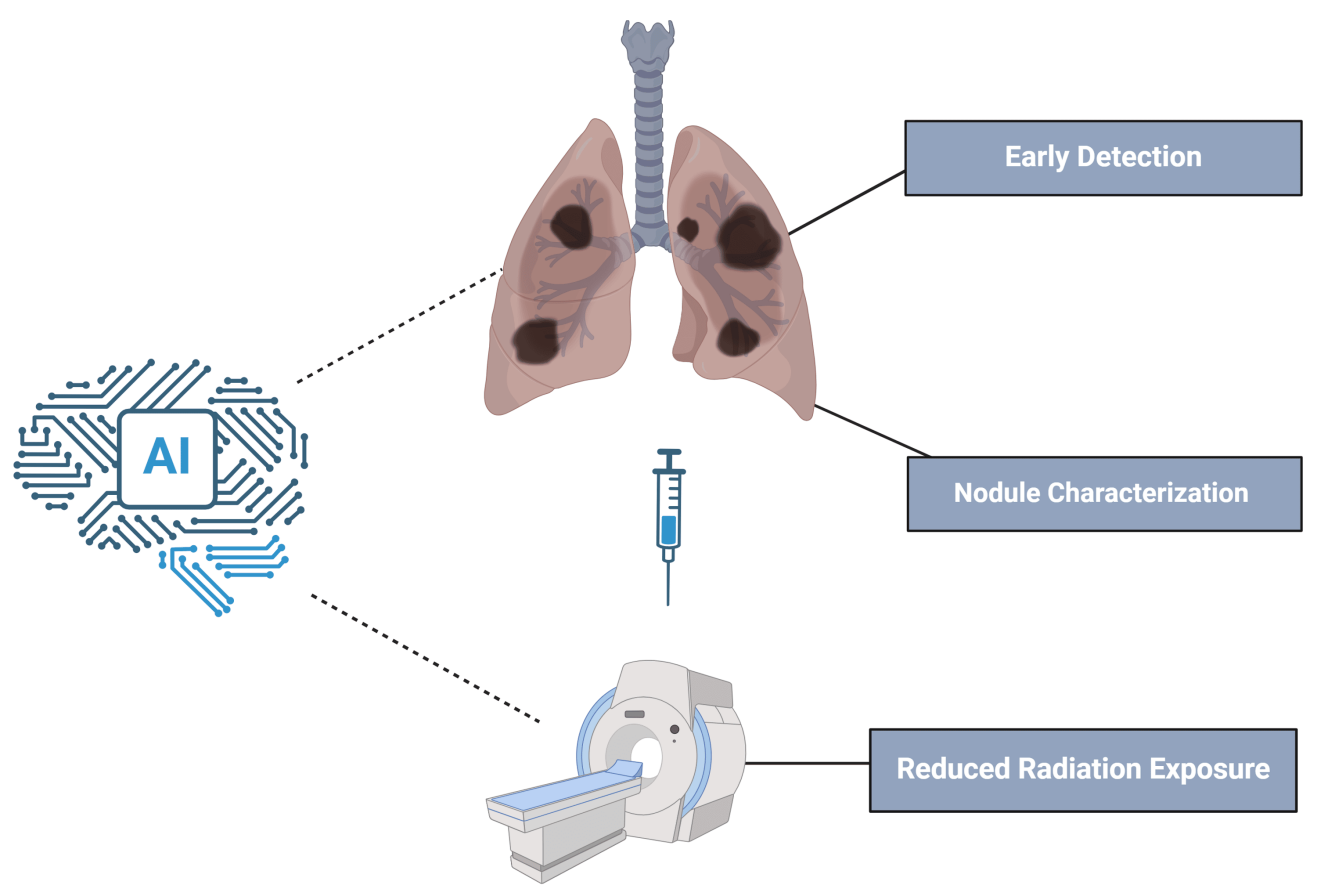
Role of AI in screening of lung cancer (Image credit: Rinkoo FNU)

AI systems employing DL have been created to identify malignant pulmonary nodules in chest CT scans, aiding physicians in enhancing the precision and efficiency of lung cancer screening. This technology has demonstrated effectiveness in differentiating CT images, thereby enhancing the efficiency of lung cancer screening (Figure 5). Diannei Technology Co. Ltd. has designed an AI diagnostic system that uses the 3D DenseSharp network, which predicts invasive labeling and lesion segmentation more accurately than radiologists [65].

Lung nodules can be classified as benign or malignant and are early indicators of lung cancer. Detecting these nodules early and accurately is crucial for improving treatment outcomes and survival rates. Radiologists evaluate the location, size, and density variations of nodules compared to nearby structures. These evaluations are subjective, and preliminary CT scans failed to detect 8.9% of lung cancer cases, according to the American Lung Cancer Screening Program [66]. 

Radiomics involves manually defining the region of interest and extracting detailed features from medical images to create data for statistical models and predictive analytics [67, 68]. Features such as histogram characteristics, size, shape, texture parameters and are frequently extracted to quantitatively and objectively define tumors and nodules [69]. Radiomic signatures are developed by integrating selected features with traditional ML methods to predict clinical outcomes. Although they have their limitations, traditional ML classifiers such as support vector machine (SVM) and random forest (RF) often provide good results. Multi-class problems and huge datasets are a real challenge for SVMs, and manual feature extraction is essential for the best results with most ML classifiers. In medical image analysis, when the diagnostic goal is difficult and previous information is restricted, this extraction procedure is labor-intensive and complicated. Despite clinicians' expertise, understanding which imaging features predict outcomes is difficult, and manually extracting lung nodule characteristics is particularly challenging.

In contrast, DL algorithms, particularly CNN learn features directly from dataset given by humans. They are highly automated and require minimal manual input, as they generate valuable representations through data-driven learning, without relying on manually collected information about lung nodules. Additionally, DL algorithms can more readily transfer knowledge from other fields to lung cancer diagnosis. The integration of radiomics with DL allows for the processing of large volumes of data and can be applied to the radiological diagnosis of diseases, including lung cancer [68, 70]. Overall, DL outperforms traditional ML and radiomics and has revolutionized medical image analysis since its breakthrough in 2012 with AlexNet.

Zhang et al. employed clinical CT scans to identify lung nodules using a three-dimensional CNN. After being trained on public LDCT photos from lung cancer screenings, the model was tested on a 50-image set including preoperative CTs and verified using clinical LDCT images from four hospitals. In terms of identifying and categorizing nodules, the DL system outperformed 25 qualified doctors [71]. Bear in mind that the validation data was sourced from a multicenter dataset with images of variable quality and a small sample size of ground-glass nodules; these factors may have affected the accuracy of the nodule categorization. Regardless, the model outperformed doctors, demonstrating the promise of DL for clinical lung cancer detection. 

Computer-aided diagnosis tools use AI algorithms to assist radiologists in detecting pulmonary nodules and reducing the false-positive rate. These systems are categorized into two types: computer-aided detection tools and computer-aided diagnosis tools. These systems use DL models, trained on large volumes of comprehensive datasets, to automate nodule detection with high sensitivity and specificity. CNN trained on public databases facilitate broader research on the subject as well as algorithm performance comparisons [72]. A recent retrospective analysis evaluated deep neural networks for lung nodule detection by comparing their performance to radiologists assessing real-world LDCT images. Trained on 39,014 chest LDCT cases and validated with 600 cases and the LUNA public dataset, the model excelled in differentiating nodule sizes and types. The results matched or surpassed radiologists in detecting both large and small nodules with superior sensitivity, ROC-AUC performance, and specificity for classifying true positives [73]. Although the study lacked details like smoking history, lung diseases, and other health conditions, the model's training on a large, multicenter clinical database suggests it can be widely applied and is likely to be effective in different settings.

AI and Chest X-Rays

LDCT scans for lung cancer screening are generally accessible for those who meet the screening criteria, with broad insurance coverage supporting this. Mobile LDCT units equipped with AI-assisted diagnostics have successfully increased access in underserved areas, such as rural China, where 67.94% of participants in a study completed screening [74]. Programs placing patient navigators in clinics have significantly increased participation rates, especially among racial and ethnic minorities [75].

Expanding insurance coverage and providing subsidies for LDCT screening in low-income populations has been proposed as a strategy to enhance uptake [76]. Training primary care physicians to proactively recommend LDCT screening and using patient education campaigns have been shown to improve screening adherence [77].

However, geographic and socioeconomic factors can negatively influence individual access. In these circumstances, chest X-rays will remain as the traditional imaging alternative. Coupling CADe and chest X-ray imaging have been developed to detect major abnormalities, including pulmonary nodules. 

AI tools have been shown to improve nodule detection accuracy in settings where radiologists are limited, bridging the diagnostic gap [78]. Many patients remain unaware of LDCT availability; studies show patient education significantly increases uptake [79]. 

Yoo et al. conducted a study on commercially available CADe tools that showed that while sensitivity and specificity for detecting nodules were similar to those of radiologists, AI improved radiologists' performance in detecting malignant nodules and reduced false-positives per radiographs when used in conjunction with radiologist readings [80]. In contrast, Sim et al.'s study demonstrates that the CAD tool significantly enhances radiologists' sensitivity and reduces false-positives; however, the tool's standalone performance was lower in sensitivity [81]. AI should function as a second reader, with final validation by an available radiologist or remote specialist. Studies show that AI-augmented readings outperform standalone AI or radiologist interpretations alone [45]. Remote AI-assisted LDCT readings with cloud-based teleconsultation allow radiologists in urban centers to provide oversight for underserved areas [74]. Implementing AI training programs for general practitioners in remote areas ensures better oversight of AI-generated findings and reduces misdiagnosis risks [82].

AI and PET scans 

The significance of AI in lung cancer imaging extends beyond tumor detection to include lung cancer staging. Passive voice to active voice - AI PET image analysis can enhance tumor staging precision. CNNs utilize multiplanar reconstruction of PET and CT scans, along with the integration of ^18^F fluorodeoxyglucose (FDG)- PET-MIP (maximum intensity projection) and atlases, to ascertain the anatomical positioning of ^18^F-FDG lesions and assess their potential malignancy. This approach sets the benchmark for training various CNNs and evaluating their effectiveness in classification and localization [65].

AI and Biomarkers

**Figure 6:**
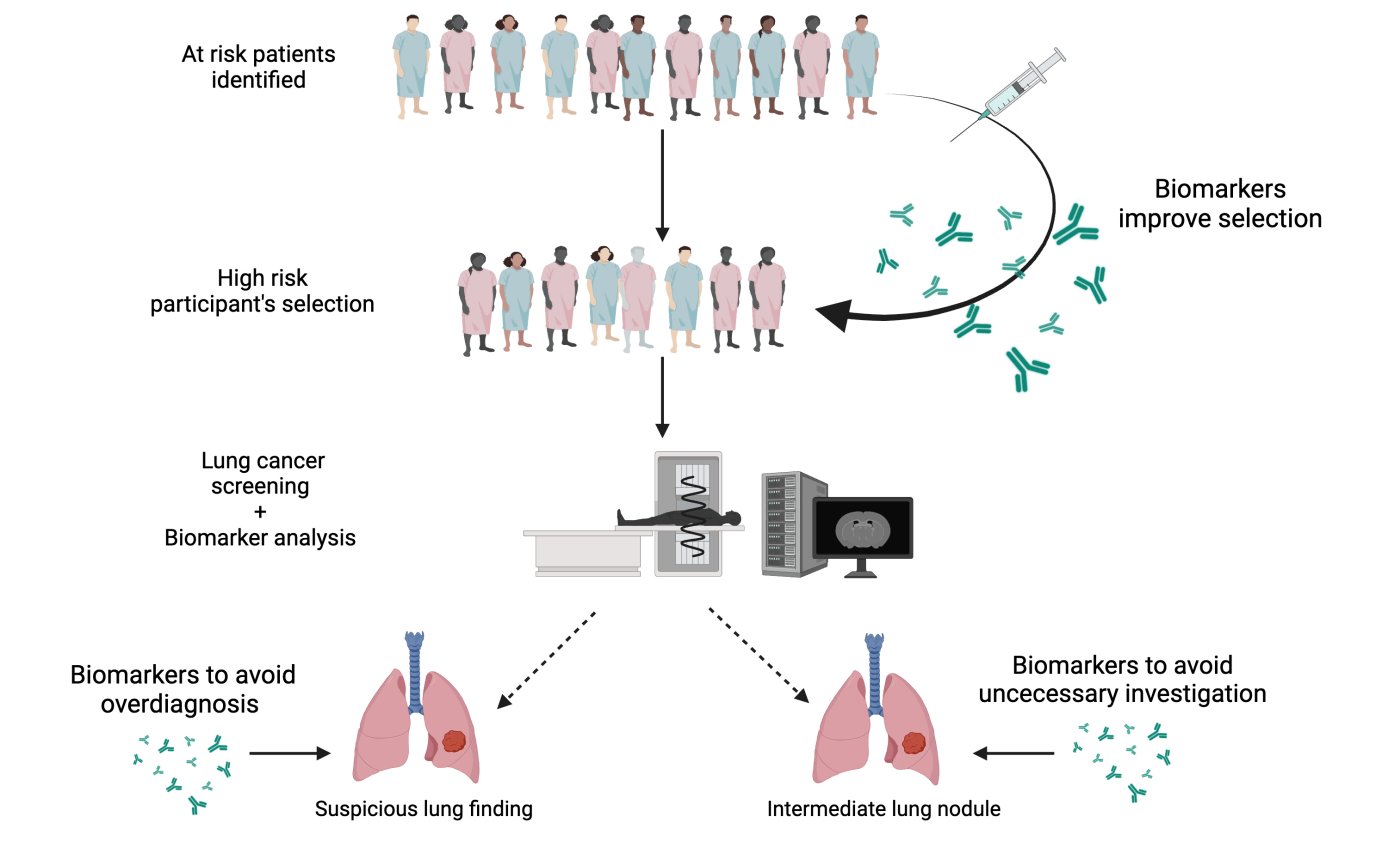
Integration of biomarkers and lung cancer screening (Image credit: Richard L. Dookie)

LDCT has a high false-positive rate but is straightforward and very sensitive for identifying lung nodules. A potential future avenue to solve this is the development of novel screening indicators, such as biomarkers supported by evidence [83]. Using a microRNA signature classifier may improve sensitivity from 84% to 98% and decrease the false-positive rate of LDCT by as much as 80%, according to research [83]. With a negative predictive value of more than 99%, serum microRNA testing allows patients to forego follow-ups if their findings come back negative. The use of serum RNA by ML models allows for the accurate prediction of lung cancer years prior to diagnosis. In the 10 years before lung cancer diagnosis, the research gathered 1061 samples from 925 individuals, with each sample undergoing an average of 18 million RNA sequencing. The average AUC for non-small-cell lung cancer (NSCLC) prediction models zero to two years before diagnosis was 0.89 (95% CI, 0.84-0.96), whereas for six to eight years before diagnosis, it was 0.82 (95% CI, 0.76-0.88) [84]. Integrating LDCT with biomarkers and AI offers the potential to enhance screening efficiency and reduce costs, although initial expenses may be high. The advancements in AI and biomarkers are expected to lead to more effective long-term outcomes (Figure 6).

Challenges and future solutions

Lack of Extensive and Accurately Labeled Datasets

Developing AI-based tools requires extensive high-quality data. Low data sharing and variability in CT image annotation standards complicate the development of robust AI models. The amount of lung cancer information is vast, but in order to build reliable algorithms, clinical and laboratory data has to be gathered in a consistent and structured manner [85]. The value of combining data from several sources emphasizes the significance of researchers working together and sharing their findings. Researches might confirm their local investigations with the use of open-access libraries like "The Cancer Imaging Archive" that provide vast cancer datasets and are constantly growing [86]. 

Another approach to dataset scarcity is data augmentation techniques like cropping, rotation, and flipping, which can increase dataset size and diversity. GANs can also create additional synthetic images to supplement existing data [87]. Advanced CNNs can be trained using semi-supervised and self-supervised learning methods on raw CT scans, potentially outperforming traditional supervised methods [88, 89]. Additionally, transfer learning can enhance nodule identification and classification accuracy by pre-training 3D CNNs on large datasets. 

Multivariable Analysis

Researchers should recognize that incorporating multiple data sources is essential for designing an AI model that fully characterizes lung cancer. Multivariate analysis, including non-imaging characteristics, such as family history, clinical information, and genetic data Is recommended for building a more comprehensive model [90].

Reproducibility 

This is a major challenge for AI in clinical settings. This is because there are differences in the radiomics procedure, which includes image capture and model validation, across studies and institutions [91]. For instance, differences in image acquisition protocols can impact signal-to-noise ratios and image characteristics, leading to variations in imaging features that might be attributed to acquisition parameters rather than actual differences in tissue properties [91]. One approach to address this issue is to exclude features significantly affected by acquisition and reconstruction parameters [92]. Another potential solution is to enhance standardization by adopting open imaging protocols.

Generalization Ability

Many DL models have been created to address various diagnostic issues. Although these models often perform exceptionally well in their targeted applications, a common problem is that models that excel in one specific task often fail to generalize to other, even slightly different tasks. Poor generalization can increase the risk of misdiagnosis and missed diagnoses, which can negatively impact patient health and the effectiveness of treatment plans. Employing multi-task learning, which allows models to perform related tasks simultaneously, can improve generalization [93]. Cloud computing can facilitate real-time updates to training datasets, helping models adapt to different scanning devices and imaging modalities [94]. The use of diverse medical scanning devices and imaging modalities can affect the generalizability of DL models. To improve model performance, it is crucial to investigate how different scanning parameters and image reconstruction techniques impact results, and then optimize the models for these various settings.

## Conclusions

AI detection models for breast, lung, and colon cancer show promise in improving diagnostic efficiency, objectivity, and reducing clinician workload, but are still in clinical exploration. To advance these models, it is crucial to assess AI systems using confirmed pathology and not just radiologists' consensus. Future multicenter studies should evaluate AI performance and how its risk scores impact radiologists' accuracy, as well as explore their integration into follow-up protocols. While AI cannot replace clinical decision-making, it is expected to support and enhance care. However, challenges remain, such as the lack of standardized datasets, ethical and legal issues, and model generalization. To realize AI's full potential, future efforts must focus on improving transparency, explainability, and data diversity, while ensuring validation in real-world settings. Addressing these challenges could revolutionize cancer screening, reduce mortality, and improve patient outcomes.

## References

[1] Robertson AR, Segui S, Wenzek H, Koulaouzidis A (2021). Artificial intelligence for the detection of polyps or cancer with colon capsule endoscopy. Ther Adv Gastrointest Endosc.

[2] Hunter B, Hindocha S, Lee RW (2022). The role of artificial intelligence in early cancer diagnosis. Cancers (Basel).

[3] Bray F, Laversanne M, Sung H, Ferlay J, Siegel RL, Soerjomataram I, Jemal A (2024). Global cancer statistics 2022: GLOBOCAN estimates of incidence and mortality worldwide for 36 cancers in 185 countries. CA Cancer J Clin.

[4] Moleyar-Narayana P, Leslie S, Ranganathan S (2024). Cancer screening. StatPearls.

[5] Noor J, Chaudhry A, Batool S (2023). Microfluidic technology, artificial intelligence, and biosensors as advanced technologies in cancer screening: a review article. Cureus.

[6] Hu Q, Giger ML (2021). Clinical artificial intelligence applications: breast imaging. Radiol Clin North Am.

[7] Li Y, Wu X, Yang P, Jiang G, Luo Y (2022). Machine learning for lung cancer diagnosis, treatment, and prognosis. Genomics Proteomics Bioinformatics.

[8] Rao HB, Sastry NB, Venu RP, Pattanayak P (2022). The role of artificial intelligence based systems for cost optimization in colorectal cancer prevention programs. Front Artif Intell.

[9] Shaukat A, Lichtenstein DR, Somers SC (2022). Computer-aided detection improves adenomas per colonoscopy for screening and surveillance colonoscopy: a randomized trial. Gastroenterology.

[10] Pecere S, Antonelli G, Dinis-Ribeiro M (2022). Endoscopists performance in optical diagnosis of colorectal polyps in artificial intelligence studies. United European Gastroenterol J.

[11] Cellina M, Cacioppa LM, Cè M (2023). Artificial intelligence in lung cancer screening: the future is now. Cancers (Basel).

[12] Elhakim MT, Graumann O, Larsen LB, Nielsen M, Rasmussen BS (2020). Kunstig intelligens til cancerdiagnostik i brystkraeftscreening [Artificial intelligence for cancer diagnostics in breast cancer screening] (in Danish). Ugeskr Laeger.

[13] Bray F, Ferlay J, Soerjomataram I, Siegel RL, Torre LA, Jemal A (2018). Global cancer statistics 2018: GLOBOCAN estimates of incidence and mortality worldwide for 36 cancers in 185 countries. CA Cancer J Clin.

[14] Sha R, Kong XM, Li XY, Wang YB (2024). Global burden of breast cancer and attributable risk factors in 204 countries and territories, from 1990 to 2021: results from the Global Burden of Disease Study 2021. Biomark Res.

[15] Mustafa M, Abbas K, Alam M (2024). Molecular pathways and therapeutic targets linked to triple-negative breast cancer (TNBC). Mol Cell Biochem.

[16] Wang X, Ahmad I, Javeed D (2022). Intelligent hybrid deep learning model for breast cancer detection. Electronics.

[17] Zhang H, Wu R, Yuan T (2020). DE-Ada*: a novel model for breast mass classification using cross-modal pathological semantic mining and organic integration of multi-feature fusions. Information Sciences.

[18] Pesapane F, Rotili A, Agazzi GM (2021). Recent radiomics advancements in breast cancer: lessons and pitfalls for the next future. Curr Oncol.

[19] Goodfellow I, Bengio Y, Courville A (2016). Deep Learning. https://mitpress.mit.edu/9780262035613/deep-learning/.

[20] Nalla V, Pouriyeh S, Parizi RM (2024). Deep learning for computer-aided abnormalities classification in digital mammogram: A data-centric perspective. Curr Probl Diagn Radiol.

[21] Al-Hejri AM, Al-Tam RM, Fazea M, Sable AH, Lee S, Al-Antari MA (2022). ETECADx: ensemble self-attention transformer encoder for breast cancer diagnosis using full-field digital X-ray breast images. Diagnostics (Basel).

[22] Nasser M, Yusof UK (2023). Deep learning based methods for breast cancer diagnosis: a systematic review and future direction. Diagnostics (Basel).

[23] Bai J, Posner R, Wang T, Yang C, Nabavi S (2021). Applying deep learning in digital breast tomosynthesis for automatic breast cancer detection: a review. Med Image Anal.

[24] Arun Kumar S, Sasikala S (2023). Review on deep learning-based CAD systems for breast cancer diagnosis. Technol Cancer Res Treat.

[25] Anderson AW, Marinovich ML, Houssami N (2022). Independent external validation of artificial intelligence algorithms for automated interpretation of screening mammography: a systematic review. J Am Coll Radiol.

[26] Zeng A, Houssami N, Noguchi N, Nickel B, Marinovich ML (2024). Frequency and characteristics of errors by artificial intelligence (AI) in reading screening mammography: a systematic review. Breast Cancer Res Treat.

[27] Díaz O, Rodríguez-Ruíz A, Sechopoulos I (2024). Artificial Intelligence for breast cancer detection: technology, challenges, and prospects. Eur J Radiol.

[28] Din NM, Dar RA, Rasool M, Assad A (2022). Breast cancer detection using deep learning: datasets, methods, and challenges ahead. Comput Biol Med.

[29] Dileep G, Gianchandani Gyani SG (2022). Artificial intelligence in breast cancer screening and diagnosis. Cureus.

[30] Gao Y, Lin J, Zhou Y, Lin R (2023). The application of traditional machine learning and deep learning techniques in mammography: a review. Front Oncol.

[31] Lopes SR, Martins C, Santos IC, Teixeira M, Gamito É, Alves AL (2024). Colorectal cancer screening: a review of current knowledge and progress in research. World J Gastrointest Oncol.

[32] Larsen SL, Mori Y (2022). Artificial intelligence in colonoscopy: a review on the current status. DEN Open.

[33] Bang CS, Lee JJ, Baik GH (2021). Computer-aided diagnosis of diminutive colorectal polyps in endoscopic images: systematic review and meta-analysis of diagnostic test accuracy. J Med Internet Res.

[34] Antonelli G, Gkolfakis P, Tziatzios G, Papanikolaou IS, Triantafyllou K, Hassan C (2020). Artificial intelligence-aided colonoscopy: recent developments and future perspectives. World J Gastroenterol.

[35] Li JW, Wang LM, Ang TL (2022). Artificial intelligence-assisted colonoscopy: a narrative review of current data and clinical applications. Singapore Med J.

[36] Khalaf K, Fujiyoshi MR, Spadaccini M (2024). From staining techniques to artificial intelligence: a review of colorectal polyps characterization. Medicina (Kaunas).

[37] Barua I, Vinsard DG, Jodal HC (2021). Artificial intelligence for polyp detection during colonoscopy: a systematic review and meta-analysis. Endoscopy.

[38] El Zoghbi M, Shaukat A, Hassan C, Anderson JC, Repici A, Gross SA (2023). Artificial intelligence-assisted optical diagnosis: a comprehensive review of its role in leave-in-situ and resect-and-discard strategies in colonoscopy. Clin Transl Gastroenterol.

[39] Gimeno-García AZ, Hernández-Pérez A, Nicolás-Pérez D, Hernández-Guerra M (2023). Artificial intelligence applied to colonoscopy: is it time to take a step forward?. Cancers (Basel).

[40] Parsa N, Byrne MF (2021). Artificial intelligence for identification and characterization of colonic polyps. Ther Adv Gastrointest Endosc.

[41] Zhang H, Yang X, Tao Y, Zhang X, Huang X (2023). Diagnostic accuracy of endocytoscopy via artificial intelligence in colorectal lesions: a systematic review and meta‑analysis. PLoS One.

[42] Kikuchi R, Okamoto K, Ozawa T, Shibata J, Ishihara S, Tada T (2024). Endoscopic artificial intelligence for image analysis in gastrointestinal neoplasms. Digestion.

[43] Baumer S, Streicher K, Alqahtani SA (2023). Accuracy of polyp characterization by artificial intelligence and endoscopists: a prospective, non-randomized study in a tertiary endoscopy center. Endosc Int Open.

[44] Pham NB, Vu KT, Nguyen NH, Doan HT, Tran TT (2022). Magnifying chromoendoscopy with flexible spectral imaging color enhancement, indigo carmine, and crystal violet in predicting the histopathology of colorectal polyps: diagnostic value in a scare-setting resource. Gastroenterol Res Pract.

[45] Djinbachian R, Haumesser C, Taghiakbari M (2024). Autonomous artificial intelligence vs artificial intelligence-assisted human optical diagnosis of colorectal polyps: a randomized controlled trial. Gastroenterology.

[46] Jha D, Sharma V, Banik D (2025). Validating polyp and instrument segmentation methods in colonoscopy through Medico 2020 and MedAI 2021 Challenges. Med Image Anal.

[47] Messmann H, Bisschops R, Antonelli G (2022). Expected value of artificial intelligence in gastrointestinal endoscopy: European Society of Gastrointestinal Endoscopy (ESGE) Position Statement. Endoscopy.

[48] Wang KW, Dong M (2020). Potential applications of artificial intelligence in colorectal polyps and cancer: recent advances and prospects. World J Gastroenterol.

[49] Joseph J, LePage EM, Cheney CP, Pawa R (2021). Artificial intelligence in colonoscopy. World J Gastroenterol.

[50] Vadhwana B, Tarazi M, Patel V (2023). The role of artificial intelligence in prospective real-time histological prediction of colorectal lesions during colonoscopy: a systematic review and meta-analysis. Diagnostics (Basel).

[51] Mehta A, Kumar H, Yazji K (2023). Effectiveness of artificial intelligence-assisted colonoscopy in early diagnosis of colorectal cancer: a systematic review. Int J Surg.

[52] Glissen Brown JR, Mansour NM, Wang P (2022). Deep learning computer-aided polyp detection reduces adenoma miss rate: a United States multi-center randomized tandem colonoscopy study (CADeT-CS trial). Clin Gastroenterol Hepatol.

[53] Keshtkar K, Safarpour AR, Heshmat R, Sotoudehmanesh R, Keshtkar A (2023). A systematic review and meta-analysis of convolutional neural network in the diagnosis of colorectal polyps and cancer. Turk J Gastroenterol.

[54] van der Zander QE, Roumans R, Kusters CH (2024). Appropriate trust in artificial intelligence for the optical diagnosis of colorectal polyps: the role of human/artificial intelligence interaction. Gastrointest Endosc.

[55] Xu L, He X, Zhou J (2021). Artificial intelligence-assisted colonoscopy: a prospective, multicenter, randomized controlled trial of polyp detection. Cancer Med.

[56] Saquib N, Saquib J, Ioannidis JP (2015). Does screening for disease save lives in asymptomatic adults? Systematic review of meta-analyses and randomized trials. Int J Epidemiol.

[57] Toumazis I, Bastani M, Han SS, Plevritis SK (2020). Risk-Based lung cancer screening: a systematic review. Lung Cancer.

[58] Frost JK, Ball WC Jr, Levin ML (1984). Early lung cancer detection: results of the initial (prevalence) radiologic and cytologic screening in the Johns Hopkins study. Am Rev Respir Dis.

[59] Melamed MR, Flehinger BJ, Zaman MB, Heelan RT, Perchick WA, Martini N (1984). Screening for early lung cancer. Results of the Memorial Sloan-Kettering study in New York. Chest.

[60] Fontana RS, Sanderson DR, Taylor WF, Woolner LB, Miller WE, Muhm JR, Uhlenhopp MA (1984). Early lung cancer detection: results of the initial (prevalence) radiologic and cytologic screening in the Mayo Clinic study. Am Rev Respir Dis.

[61] Kubik A, Polak J (1986). Lung cancer detection results of a randomized prospective study in Czechoslovakia. Cancer.

[62] Kaneko M, Eguchi K, Ohmatsu H, Kakinuma R, Naruke T, Suemasu K, Moriyama N (1996). Peripheral lung cancer: screening and detection with low-dose spiral CT versus radiography. Radiology.

[63] Sone S, Takashima S, Li F (1998). Mass screening for lung cancer with mobile spiral computed tomography scanner. The. Lancet.

[64] Aberle DR, Adams AM, Berg CD (2011). Reduced lung-cancer mortality with low-dose computed tomographic screening. N Engl J Med.

[65] Pei Q, Luo Y, Chen Y, Li J, Xie D, Ye T (2022). Artificial intelligence in clinical applications for lung cancer: diagnosis, treatment and prognosis. Clin Chem Lab Med.

[66] Scholten ET, Horeweg N, de Koning HJ, Vliegenthart R, Oudkerk M, Mali WP, de Jong PA (2015). Computed tomographic characteristics of interval and post screen carcinomas in lung cancer screening. Eur Radiol.

[67] Chassagnon G, Vakalopolou M, Paragios N, Revel MP (2020). Deep learning: definition and perspectives for thoracic imaging. Eur Radiol.

[68] Fornacon-Wood I, Faivre-Finn C, O'Connor JP, Price GJ (2020). Radiomics as a personalized medicine tool in lung cancer: separating the hope from the hype. Lung Cancer.

[69] Nakaura T, Higaki T, Awai K, Ikeda O, Yamashita Y (2020). A primer for understanding radiology articles about machine learning and deep learning. Diagn Interv Imaging.

[70] Koçak B, Durmaz EŞ, Ateş E, Kılıçkesmez Ö (2019). Radiomics with artificial intelligence: a practical guide for beginners. Diagn Interv Radiol.

[71] Zhang C, Sun X, Dang K (2019). Toward an expert level of lung cancer detection and classification using a deep convolutional neural network. Oncologist.

[72] Setio AA, Traverso A, de Bel T (2017). Validation, comparison, and combination of algorithms for automatic detection of pulmonary nodules in computed tomography images: the LUNA16 challenge. Med Image Anal.

[73] Cui S, Ming S, Lin Y (2020). Development and clinical application of deep learning model for lung nodules screening on CT images. Sci Rep.

[74] Tao W, Yu X, Shao J, Li R, Li W (2024). Telemedicine-enhanced lung cancer screening using mobile computed tomography unit with remote artificial intelligence assistance in underserved communities: initial results of a population cohort study in western China. Telemed J E Health.

[75] Khan H, Ramphal K, Motia M (2023). Disparities in lung cancer screening in a diverse urban population and the impact of a community-based navigational program. Journal of Clinical Oncology.

[76] Presant CA, Ashing K, Raz D (2023). Overcoming barriers to tobacco cessation and lung cancer screening among racial and ethnic minority groups and underserved patients in academic centers and community network sites: the city of hope experience. J Clin Med.

[77] Bernstein E, Bade BC, Akgün KM, Rose MG, Cain HC (2022). Barriers and facilitators to lung cancer screening and follow-up. Semin Oncol.

[78] Osarogiagbon RU, Yang PC, Sequist LV (2023). Expanding the reach and grasp of lung cancer screening. Am Soc Clin Oncol Educ Book.

[79] Zarinshenas R, Amini A, Mambetsariev I, Abuali T, Fricke J, Ladbury C, Salgia R (2023). Assessment of barriers and challenges to screening, diagnosis, and biomarker testing in early-stage lung cancer. Cancers (Basel).

[80] Yoo H, Kim KH, Singh R, Digumarthy SR, Kalra MK (2020). Validation of a deep learning algorithm for the detection of malignant pulmonary nodules in chest radiographs. JAMA Netw Open.

[81] Sim Y, Chung MJ, Kotter E (2020). Deep convolutional neural network-based software improves radiologist detection of malignant lung nodules on chest radiographs. Radiology.

[82] Lowenstein M, Karliner L, Livaudais-Toman J (2022). Barriers and facilitators to lung cancer screening: a physician survey. Am J Health Promot.

[83] Peled N, Ilouze M (2015). Screening for lung cancer: what comes next?. J Clin Oncol.

[84] Montani F, Marzi MJ, Dezi F (2015). miR-Test: a blood test for lung cancer early detection. J Natl Cancer Inst.

[85] Sourlos N, Wang J, Nagaraj Y, van Ooijen P, Vliegenthart R (2022). Possible bias in supervised deep learning algorithms for CT lung nodule detection and classification. Cancers (Basel).

[86] Clark K, Vendt B, Smith K (2013). The Cancer Imaging Archive (TCIA): maintaining and operating a public information repository. J Digit Imaging.

[87] Han C, Kitamura Y, Kudo A (2019). Synthesizing diverse lung nodules wherever massively: 3D multi-conditional GAN-based CT image augmentation for object detection.

[88] Zhou Z, Sodha V, Siddiquee MM, Feng R, Tajbakhsh N, Gotway MB, Liang J (2019). Models genesis: generic autodidactic models for 3D medical image analysis. Med Image Comput Comput Assist Interv.

[89] Hussein S, Kandel P, Bolan CW, Wallace MB, Bagci U (2019). Lung and pancreatic tumor characterization in the deep learning era: novel supervised and unsupervised learning approaches. IEEE Trans Med Imaging.

[90] Lambin P, Leijenaar RT, Deist TM (2017). Radiomics: the bridge between medical imaging and personalized medicine. Nat Rev Clin Oncol.

[91] Christie JR, Lang P, Zelko LM, Palma DA, Abdelrazek M, Mattonen SA (2021). Artificial intelligence in lung cancer: bridging the gap between computational power and clinical decision-making. Can Assoc Radiol J.

[92] Rizzo S, Botta F, Raimondi S, Origgi D, Fanciullo C, Morganti AG, Bellomi M (2018). Radiomics: the facts and the challenges of image analysis. Eur Radiol Exp.

[93] Kim BC, Yoon JS, Choi JS, Suk HI (2019). Multi-scale gradual integration CNN for false positive reduction in pulmonary nodule detection. Neural Netw.

[94] Masood A, Yang P, Sheng B (2020). Cloud-based automated clinical decision support system for detection and diagnosis of lung cancer in chest CT. IEEE J Transl Eng Health Med.

